# The Triaxial Test of Polypropylene Fiber Reinforced Fly Ash Soil

**DOI:** 10.3390/ma15113807

**Published:** 2022-05-26

**Authors:** Lihua Li, Xin Zhang, Henglin Xiao, Jiang Zhang, Na Chen, Wentao Li

**Affiliations:** School of Civil Engineering, Architecture and Environment, Hubei University of Technology, Wuhan 430068, China; lilihua466@163.com (L.L.); xin_z@hbut.edu.cn (X.Z.); zj1208705765@163.com (J.Z.); cn_research@hbut.edu.cn (N.C.); wli20201027@hbut.edu.cn (W.L.)

**Keywords:** triaxial test, polypropylene fiber, coal fly ash, shear strength, energy absorption capacity

## Abstract

Recently, soil reinforcement using arranged or randomly distributed fibers has attracted increasing attention in geotechnical engineering. In this study, polypropylene (PP) fibers with three lengths (6, 12, and 24 mm) and three mass percentages (0.5%, 1%, and 1.5%) were used to reinforce a coal fly ash soil (FAS) mixture. Unconsolidated, undrained triaxial tests were carried out in order to study the mechanical properties of the polypropylene fiber-reinforced FAS mixture and evaluate the impact of fiber on the shear strength of the FAS mixture. It is found that the fiber length of 12 mm could significantly improve the shear strength of the polypropylene fiber reinforced FAS mixture, and little effect is shown on the shear strength while using a fiber length of 24 mm. Additional fibers enhance the energy absorption capacity of the FAS specimens and therefore the highest energy absorption capacity occurs when the fiber content is 1% and the fiber length is 12 mm. The peak deviator stress enhances impressively with the addition of polypropylene fiber. The impact of fiber on the peak deviator stress is the largest when fiber content is within 1.0%. The fiber length has little effect on the peak deviator stress when it exceeds 12 mm.

## 1. Introduction

Coal is increasingly used as an energy raw material in countries such as Russia, Australia, South Africa, etc., and fly ash is produced during the combustion of coal. However, fly ash is challenging to manage and poses adverse impacts on the environment [1]. Disposal of fly ash remains a problem, and only half of the fly ash is used worldwide [2]. Cenospheres form the main component of coal fly ash; the way cenospheres are created depends on the compounds in the coal and the systematic progress of coal combustion. The cenospheres have great engineering properties such as a smooth surface, micro particle size, small internal specific surface area, light specific gravity, and little water absorption [3,4]. Numerous studies have investigated the conversion of fly ash into a functional and valuable product. Cenospheres are considered desirable materials for applications in biomedical engineering [5,6]. Mechanical properties and acoustic applications of new epoxy-based matrix composites mixed with cenospheres were also investigated [7,8]. Coal combustion by-products and coal fly ash can also be utilized in the mining industry for mine site rehabilitation purposes and to improve the safety of miners [9,10].

The research into fly ash as an admixture with soil for geotechnical utilization has been broadly studied globally; easy availability and active physical properties can make fly ash soil cost-effective [11,12]. It is beneficial in improving engineering performance and subgrade soils [13,14]. Coal fly ash contains abundant active Silica, Alumina, Ferric oxide, and Calcium oxide, in which a train of chain hydration reaction occurs when water is affiliated [15]. The admixture of fly ash remarkably enhances the durability and stiffness of cured soils. These studies have shown that uniformly distributed fly ash completes the grading distribution of poorly graded soil and results in greater strength values [13,14,16]. However, fly ash cannot effectively improve the crack resistance and compressive strength of subgrade soil [14,17]. The occurrence of longitudinal and transverse cracks in fly ash reinforced soils have been observed in previous works [18,19]. Therefore, it is necessary to seek a method to avoid cracks occurring in fly ash-reinforced soils.

Fiber has been used as reinforcement material and has received increasing attention over recent decades; a composite material compound of polymer and fly ash is utilized to investigate the mechanical properties, fly ash surface modification, and geopolymerisation [5,6,20,21]. In this direction, numerous studies have been conducted on fiber-reinforced soil; these researchers showed that changes in the content and the length of fibers in the soil can positively affect resisting tensile cracking, bearing capacity, tensile strength, and shear strength [22,23,24]. Another study has shown that fiber-reinforced soil depicts higher resistance over the freeze-thaw reaction [25]. The reinforced soil is an isotropic material. Based on the advantages of good dispersion and easy fiber mixing, the even mixture that mixes with fiber filaments and soils is usually regarded as a homogeneous isotropic material [26]. Zhang et al. [27] reported that polypropylene fiber was incredibly influential in improving the resistance to tensile cracking of soil, and it can still bear the static load and hold the integrity of soil after dynamic failure. Cai et al. [28] found that fiber-reinforced soil took on a strain-hardening ductile failure in the compression test instead of strain-softening ductile failure that happened on plain soil. Several types of randomly oriented discrete fibers (i.e., natural, corban, steel, and synthetic) [26,29,30,31,32] are mixed with soils, sands, cement, or fly ash in a traditional way to find the geotechnical behavior of mixtures [33,34,35,36]; these studies have concluded that polypropylene fibers are good candidates for limiting crack propagation in soils and afford more significant residual strength [20].

Polypropylene fiber has been recommended as an efficient reinforcement material due to the increasing amount of plastic waste and its ready availability, unlike carbon or steel fiber, to solve the crack issue in coal fly ash soil (FAS) mixtures [16,21,37,38]. In spite of the fact that properties of fiber-reinforced FAS mixtures have been investigated by some studies [4,28,34,38], the coupling mechanism of the role of fibers in improving the mechanical property of FAS materials is still unclear. Based on previous studies, polypropylene fiber was introduced into FAS mixtures to change the brittle behavior to a more ductile one and restrict the propagation of cracks. In this study, a number of unconsolidated undrained (UU) triaxial tests have been conducted to obtain knowledge on how the strength of fiber-reinforced soil is affected by various fiber lengths, fiber content, and confining pressures. The influence of various factors on the fiber-reinforced soil materials is quantified by the present study. In geotechnical engineering, the present study of polypropylene fiber reinforced FAS mixture provides theoretical support and reference for a broad application.

## 2. Testing Materials, Apparatus, and Scheme

### 2.1. Testing Materials

**Figure 1:**
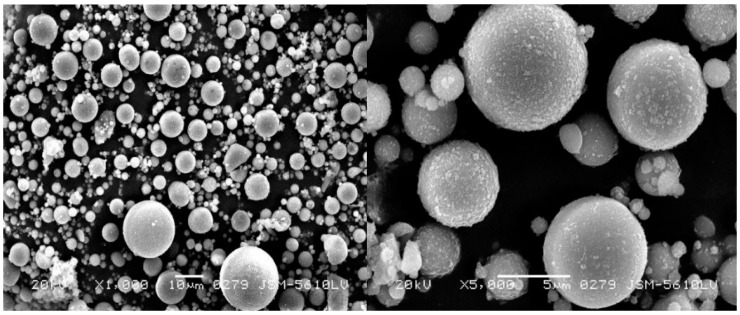
Scanning electron micrograph of coal fly ash.

**Figure 2:**
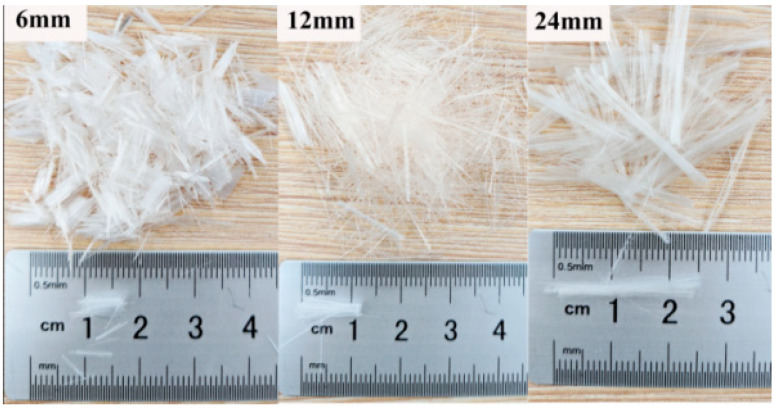
Polypropylene (PP) fiber used for the reinforcement.

The clay and fly ash utilized in this study were derived from Wuhan, Hubei Province, China. The crushed soils were sieved and collected with a size of 2 mm and below as test materials in accordance with GB/T 50123-2019 [39]. The physical properties and chemical composition of clay and fly ash are presented in Table 1 and Table 2. The clay was taken from Hubei University of Technology in Wuhan. The fly ash was a solid waste from the facility plant in Wuhan, and its chemical composition is listed in Table 2. Figure 1 presents the SEM imaging of fly ash; meanwhile, the fly ash was categorized as class C fly ash according to ASTM C618 [40]. Discretely distributed polypropylene (PP) fiber (as shown in Figure 2) that possessed a diameter of 0.023 mm was used as the reinforcement, the mechanical properties of which are recorded in Table 3.

### 2.2. Specimen Preparation

**Figure 3:**
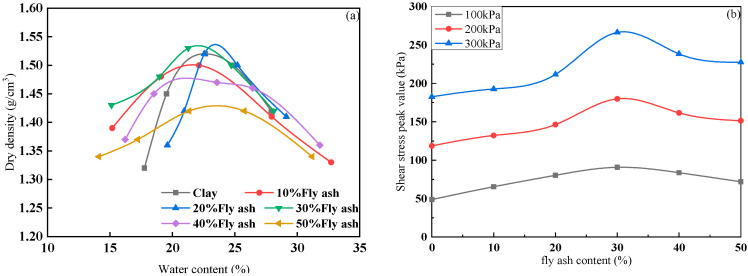
(**a**) Standard compaction curves and (**b**) Relation of shear stress peak value and fly ash content under different normal stress.

Specimens were prepared as recommended by [41]. A previous study [42] showed the standard compaction curves of the fly ash in the range of 0, 10%, 20%, 30%, 40%, and 50%, and a comparison of the optimum moisture content and maximum dry unit weight of the FAS mixtures are shown in Figure 3. The direct shear tests showed that the shear strength reached a maximum when the fly ash content was 30%, shown in Figure 3b. Therefore, the optimum proportion of fly ash to clay ratio of 30% was maintained in the present study. The optimum moisture content for FAS according to JTG E40-2007 [41] was 21.5%. The 0.5%, 1%, and 1.5% of PP fiber contents (FC) and 6, 12, and 24 mm of PP fiber length (FL) were used to prepare PP fiber-reinforced FAS specimens. The FAS and PP fiber were mixed carefully to ensure the soil samples were homogeneous [26]. Then it was followed by spraying distilled water on the samples to reach the optimal water content (21.5%). FAS mixtures are compressed with a static compactor to obtain testing specimens with a relative density of 90%, height of 300 mm, and diameter of 150 mm. Subsequently, at room temperature, all examples were sealed for 24 h to guarantee the homogeneity of water and cured for 7 days before the triaxial testing. Table 4 depicts FAS specimens mixed with PP fibers.

### 2.3. Testing Scheme

**Figure 4:**
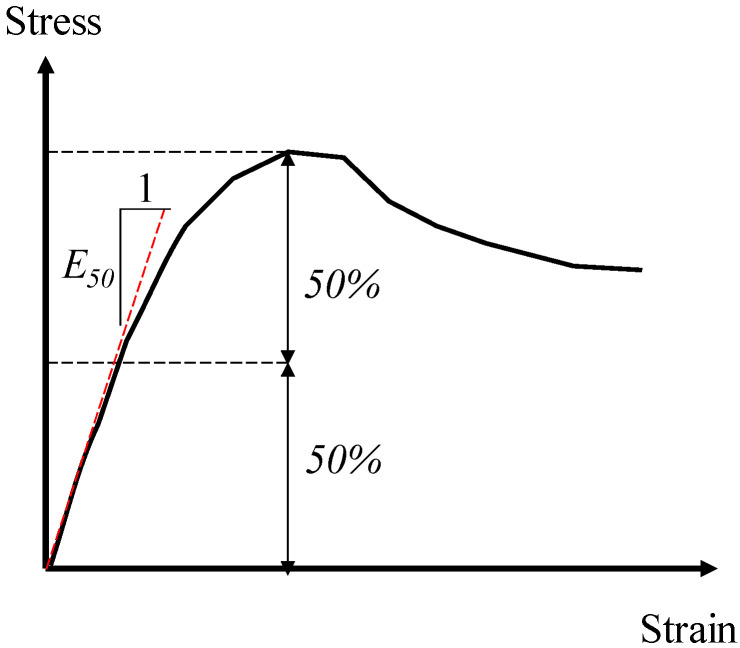
The definition of Young’s modulus, $E_{50}$.

In this study, according to ASTM D4767-11 [43], the UU triaxial test was performed on all specimens following the specific procedures. In search of the shear strength of the fiber-reinforced FAS specimens, confining pressures of 200, 300, and 400 kPa were chosen [30]. Then, the application of a strain rate of 1.5 mm/min was carried out on specimens until the axial strain reached 20%. During testing, shear stress and the axial strain were recorded. The parameter $E_{50}$ (as shown in Figure 4), which was defined as the secant modulus corresponding to 50% strength of soils in the nonlinear stress–strain curve, was adopted to evaluate the stiffness of soils [44,45].

## 3. Test Results and Discussions

### 3.1. Stress–Strain Behavior of Polypropylene Fiber-Reinforced FAS Specimens

**Figure 5:**
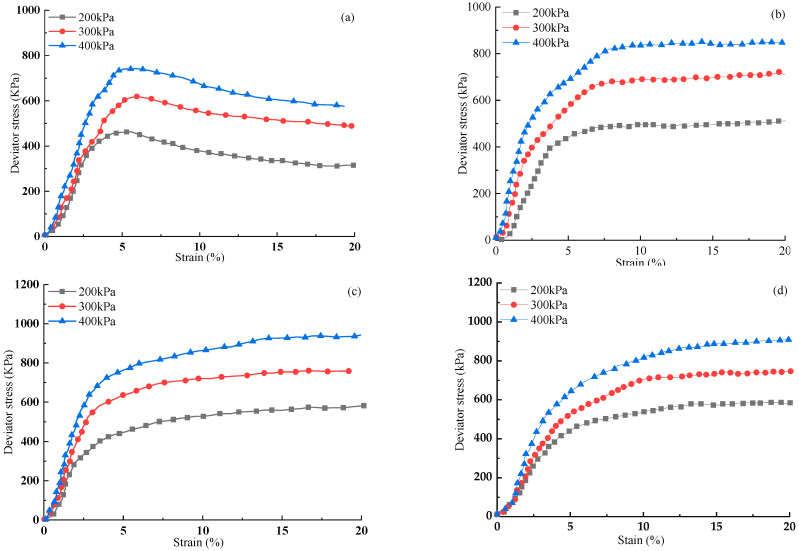
The relation of stress–strain curves of (**a**) unreinforced specimen and FAS mixtures with 0.5% FC and: (**b**) FL = 6 mm, (**c**) FL = 12 mm, (**d**) FL = 24 mm under different confining pressures.

Figure 5 appears the stress-strain curve of unreinforced specimens and FAS specimens reinforced by PP fiber.

It is shown that all non-reinforced specimens exhibit strain-softening behavior in the stress–strain relationship when the confining pressure ranges from 200 to 400 kPa. For non-reinforced specimens, the deviatoric stress increases to the peak value when axial strain is around 5%. Afterward, there is a drop when the axial strain continuously increases. For reinforced specimens (i.e., Figure 5b–d), the curves had experienced a rapid growth stage in which deviator stress increased sharply before the axial strain reached 3%, and then a placid increment known as stable stage was present after 3%. The maximum deviatoric stress enlarges with the rise of confining pressure for reinforced fibers of the same size. In contrast, all fiber-reinforced FAS specimens demonstrate the performance of strain hardening. The deviator stress of fiber-reinforced FAS increments with the rise of axial strain. Under fiber reinforcement of the same size, the deviator stress in the stable phase increases along with the rise of confining pressure, as demonstrated in Figure 5b–d. Hence, compared to plain soil, the maximum deviator stress of the fiber-reinforced FAS specimens occurs when the axial strain reaches 20%. Meanwhile, under the same fiber size and axial strain, with the rise of confining pressure, the deviator stress increases significantly. However, the cured soil after fiber reinforcement changes from the original strain softening to strain hardening. After the rapid increase of 3% strain, the strength of the soil increases slowly and changes into a strain hardening model. The ability of the soil to resist shear stress also increased significantly, indicating that the additional fibers can considerably improve the shear resistance of FAS.

**Figure 6:**
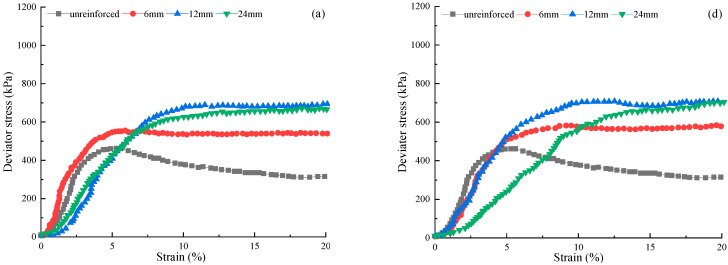

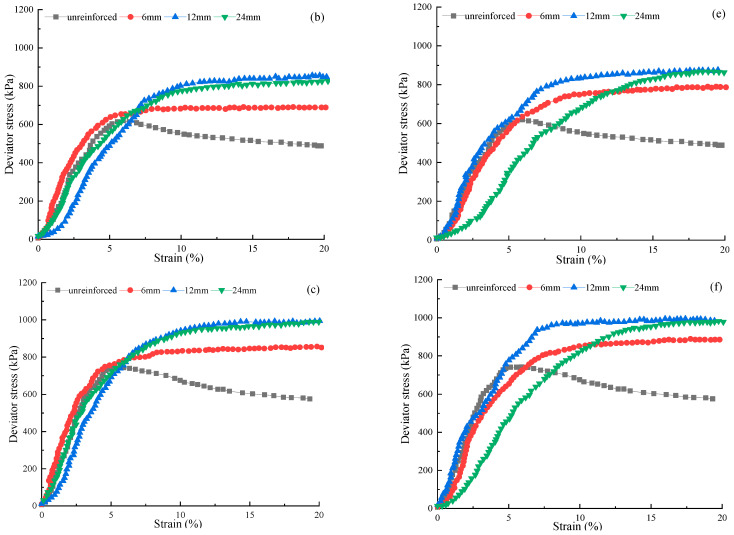
The relation of stress–strain curves of polypropylene (PP) fiber-reinforced FAS specimens: (**a**) FC = 1.0%, FL = 6 mm under 200 kPa, (**b**) FC = 1.0%, FL = 12 mm under 300 kPa, (**c**) FC = 1.0%, FL = 24 mm under 400 kPa, (**d**) FC = 1.5%, FL = 6 mm under 200 kPa, (**e**) FC = 1.5%, FL = 12 mm t under 300 kPa, (**f**) FC = 1.5%, FL = 24 mm under 400 kPa.

Figure 6 outlines the relationship between the deviator stress and the axial strain for the FAS mixtures with FC of 1%, 1.5%, and FL of 6 mm, 12 mm, and 24 mm. The curves of non-reinforced soil specimens are also provided as a comparison. For different fiber lengths, the results show that the shear stress of soil specimens increments with the size of strain, and the phenomenon of strain hardening becomes more and more prominent (from Figure 6a–c) with the rise of confining pressure. This may be because the FL 6 mm PP fibers are too short to form a more robust structure. The FAS specimens with FL 12 mm and 24 mm fibers depict a phenomenon that shear strength is not increased with the large fiber length in the end [37,46]. For example, under 200 kPa confining stress and FC 1%, the deviator stress increases from 539.6 to 689.4 kPa as the FL is elongated from 6 mm to 12 mm. When the FL increases from 12 mm to 24 mm, the deviator stress is reduced by 2.7%, and the value decreases from 689.4 to 670.6 kPa.

### 3.2. Influence of Polypropylene Fiber on Secant Modulus of FAS Specimens

**Figure 7:**
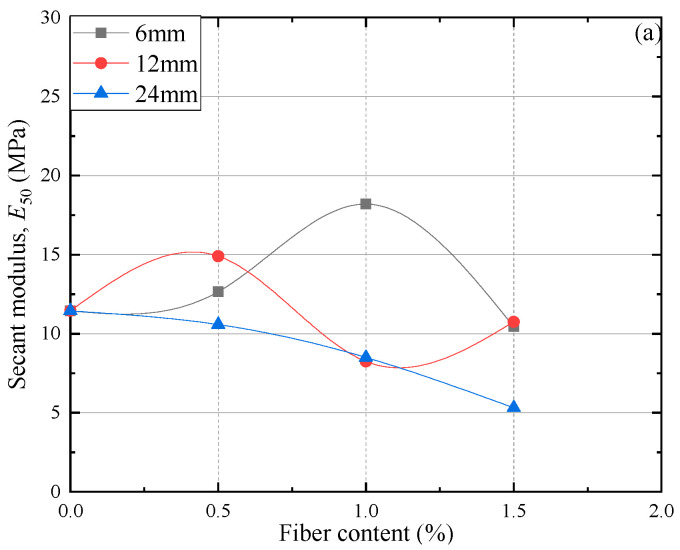

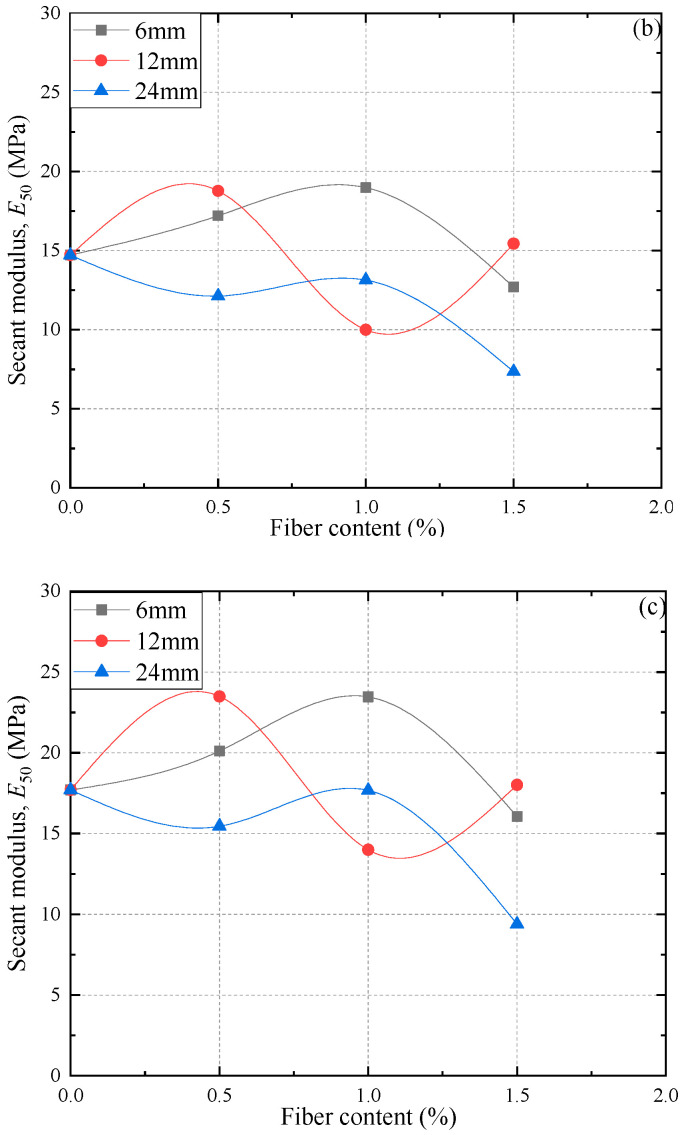
The relation curves of different FC, FL, and secant modulus, $E_{50}$, for the FAS specimens under various confining pressures: (**a**) 200 kPa, (**b**) 300 kPa, (**c**) 400 kPa.

The modulus, $E_{50}$, is an important parameter to describe the stiffness of materials [47]. The impact of the fiber length and proportion on the secant modulus is shown in Figure 7.

Figure 7 demonstrates that values of $E_{50}$ are in the scope of 11.44 MPa to 17.70 MPa for the non-reinforced FAS specimens, but in the scope of 5.31 MPa to 23.49 MPa for the PP fiber-reinforced FAS specimens. Figure 7 clearly shows that considering different deviator stresses, FC, and FL, the value of $E_{50}$ needs to be blended reasonably according to the above parameters to achieve the maximum value. At 400 kpa, the $E_{50}$ of the specimen shows the maximum value when the FC is 0.5%, and the FL is 12 mm, which is 23.49 Mpa. Afterward, the $E_{50}$ value shows a downward trend with fiber length and fiber content rise. In spite of the fact that the polypropylene fiber has a positive influence on the stiffness of soils, the connection between fly ash and soil particles may be harmed if the fiber content is higher and fiber length is longer and may further weaken the positive impact of the polypropylene fiber [48]. Therefore, there is a crucial fiber content and fiber length of the stiffness of FAS mixtures, which is of instructive significance for its application.

### 3.3. Influence of Polypropylene Fiber on the Peak Deviator Stress of Fiber-Reinforced-FAS Specimens

**Figure 8:**
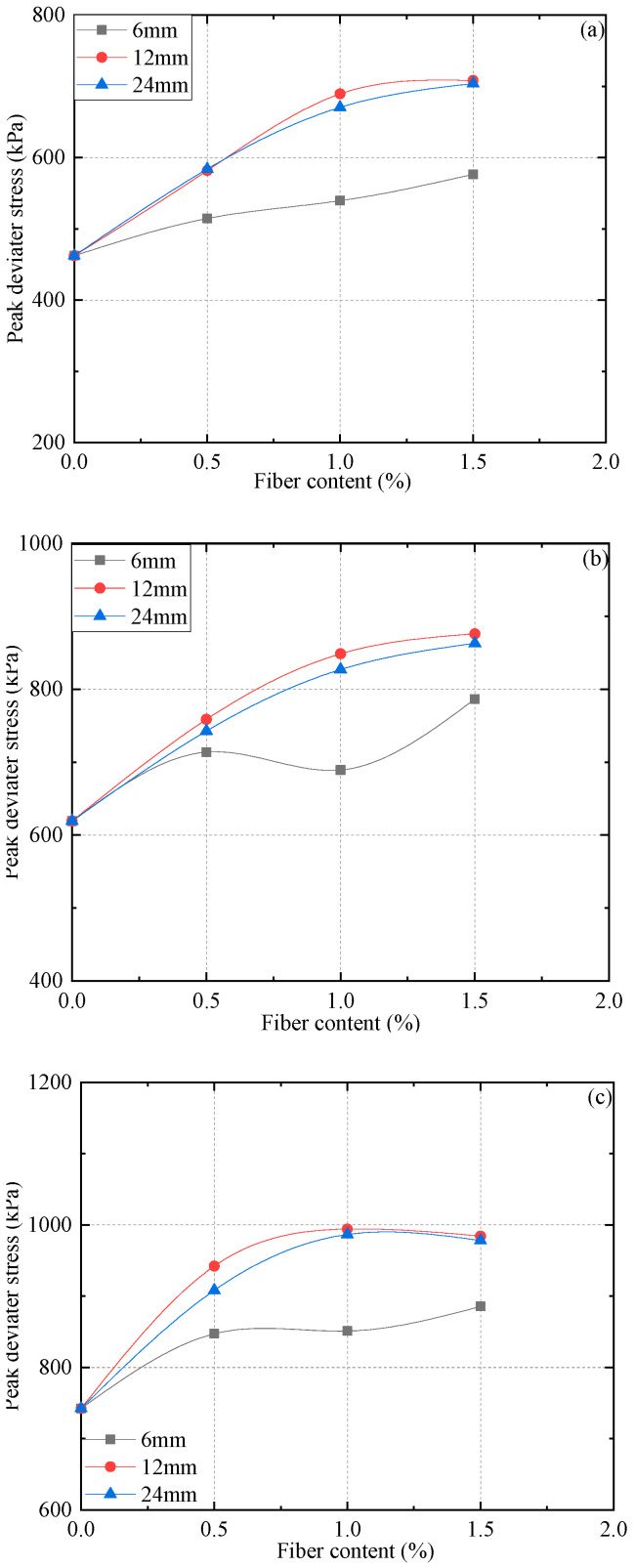
Effects of different FL and FC on the peak deviator stress of FAS specimens: (**a**) 200 kPa, (**b**) 300 kPa, (**c**) 400 kPa.

Figure 8 shows the peak deviatoric stress changes of reinforced FAS specimens with different fiber contents (0, 0.5%, 1%, and 1.5%) and different confining pressures (200 kPa, 300 kPa, and 400 kPa).

The results show that the peak deviator stress ($q_{f}$) of all reinforced FAS specimens with fibers (6, 12, and 24 mm) slightly rises with the fiber content. This is because, with the rise of fiber content, the anchorage by soil particles increases, and the interlock function between PP fibers and soil particles is closer. Normally, the peak deviator stress ($q_{f}$) increases significantly with the addition of PP fiber. Nevertheless, it can be found that when 24 mm PP fiber within the FC exceeds 0.5%, the growth rate drops considerably; when the FC is greater than 1%, the growth rate is still lower than 0.5%. The influence of fiber content on the peak deviator stress ($q_{f}$) also varies for fiber-reinforced FAS specimens with different fiber lengths: (a) with regard to the soil specimen including 6 mm in the fiber length under confining pressure of 200 kPa, the curve increases linearly from 514.6 kPa to 576.6 kPa, and the growth rate increases to 12%; (b) PP fiber-reinforced FAS specimens including 12 mm and 24 mm in the fiber lengths show a noticeable difference: the peak deviator stress both increases in the first stage, and then the increase rate has a sharp decline as the increment of fiber content rises. To summarise, the FC within 1.0% holds a remarkable impact on peak deviator stress.

Figure 8 also presents the variation in the peak deviator stress ($q_{f}$) of PP fiber-reinforced FAS specimens once the fiber length rises from 6 mm to 24 mm. It is found that there is a decreasing tendency for the peak deviator stress when the fiber length is larger than 12 mm. For example, under confining stress of 200 kPa with the FC 1.5% and as the increment of the FL rises from 6.0 mm to 12 mm, the peak deviator stress of (PP) fiber-reinforced FAS specimens rises from 576.6 kPa to 708.4 kPa; meanwhile, with the increase of FL from 12 mm to 24 mm, the peak deviator stress diminishes from 708.4 kPa to 704.1 kPa. Hence, the fiber length would have little impact on the peak deviator stress when it exceeds 12 mm.

### 3.4. Reinforcement Coefficient of Polypropylene Fiber-Reinforced FAS Specimens

In order to better describe the impact of fiber on the shear strength of FAS specimens, a parameter termed reinforcement coefficient $R_{\sigma }$ is introduced in this study and defined as:

(1) $$R_{\sigma }=\frac{q_{f,r}-q_{f,n}}{q_{f,n}},$$

where $q_{f,n}$ and $q_{f,r}$ represent the peak deviator stress of non-reinforced and reinforced specimens, respectively.

**Figure 9:**
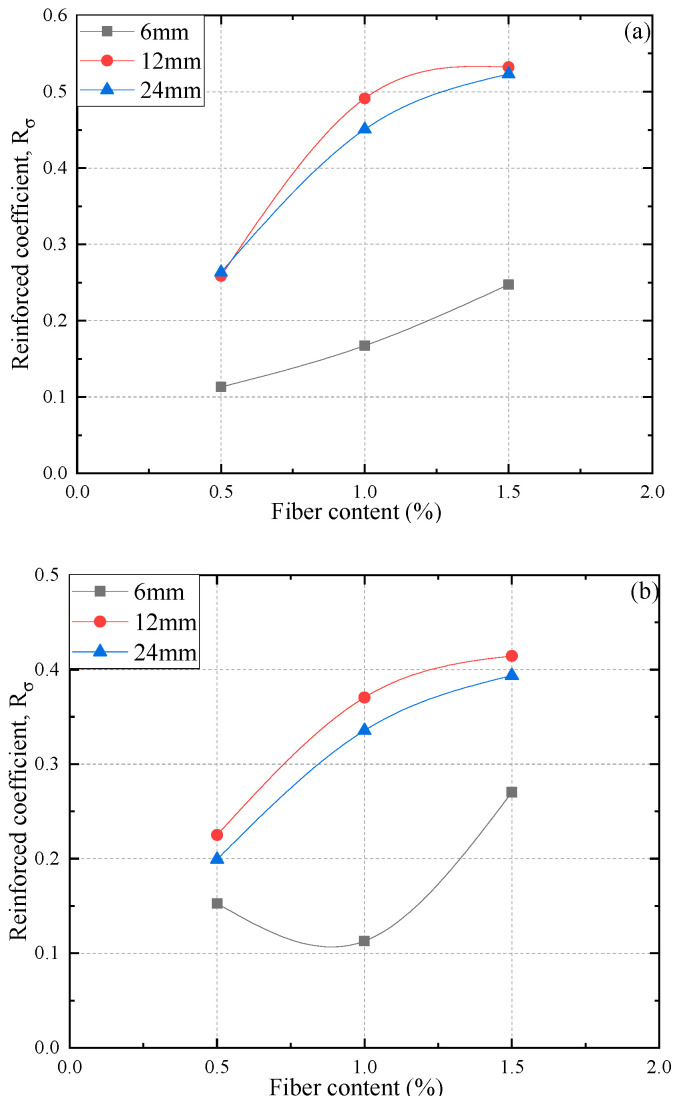

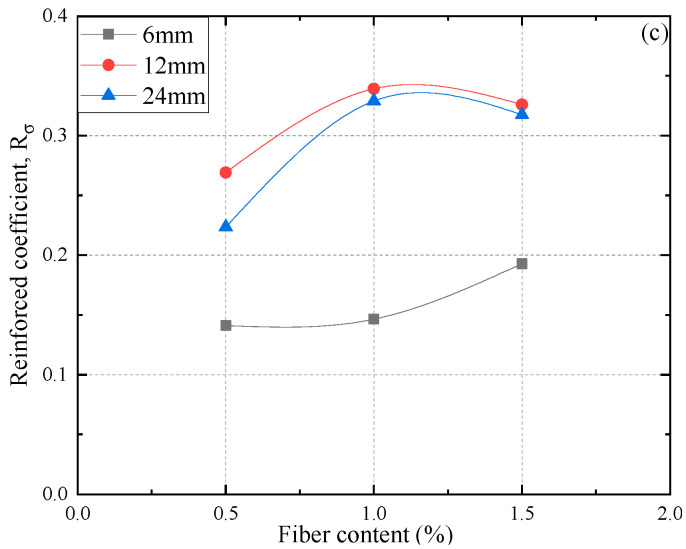
Relations between reinforced coefficient and different FC and fiber length: (**a**) under 200 kPa, (**b**) under 300 kPa, (**c**) under 400 kPa.

Figure 9 presents the $R_{\sigma }$ for all PP fiber-reinforced FAS specimens. It is clear that the values of $R_{\sigma }$ for all PP fiber-reinforced FAS specimens have an increasing tendency, indicating that fiber will result in an apparent increase within the shear strength of FAS specimens. As observed, the reinforced FAS that reaches the highest value is with FL of 12 mm and FC of 1.5% among all specimens, which results in the best performance of the reinforced FAS ground.

### 3.5. Influence of Polypropylene Fiber on the Unconsolidated Undrained Shear Strength

**Figure 10:**
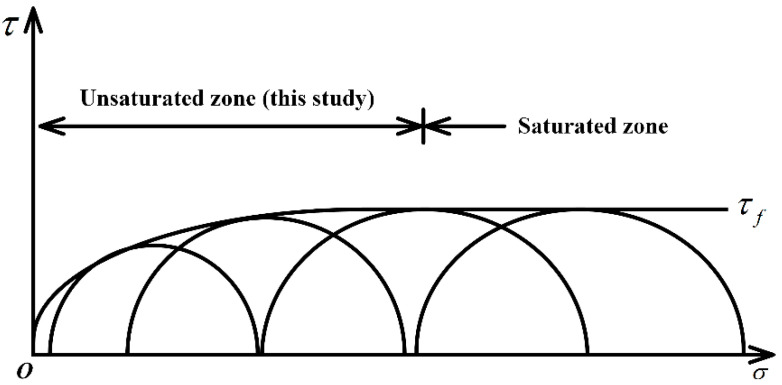
Consolidation undrained strength envelope of unsaturated clay.

Under the unconsolidated undrained experiment condition, the effective stress circles of the saturated soil are overlapped with each other, which implies that the strength envelope of the total stress circle is a horizontal line (as shown in Figure 10) [46]. The UU triaxial test results of saturated soil are usually analyzed by the Mohr–Coulomb strength theory, and the intercept of the strength envelope on the vertical axis is the shear strength, termed $c_{u}$. However, due to the saturation degree being less than 100% (unsaturated soil), and there is an error, the failure envelope of shear strength should not be horizontal. Thus, the internal friction angle $\varphi_{u}$ is not 0.

Two methods will be adopted to evaluate the shear strength parameters of soils (the cohesion $c_{uu}$ and the total angle of shearing resistance $\varphi_{uu}$). Firstly, the common tangent of the Mohr circle under different confining pressures is obtained, and the strength parameters of soils are determined via the application of the strength envelope. The other method is to use the *p*-*q* diagram of soils to calculate soil strength parameters [38,49]. In this paper, the shear strength values of PP fiber-reinforced FAS specimens are determined by the *p*-*q* diagram method. Parameters of p and q are defined as:

(2) $$p=\frac{\sigma_{1}+\sigma_{2}}{2},$$

(3) $$q=\frac{\sigma_{1}-\sigma_{2}}{2},$$

**Figure 11:**
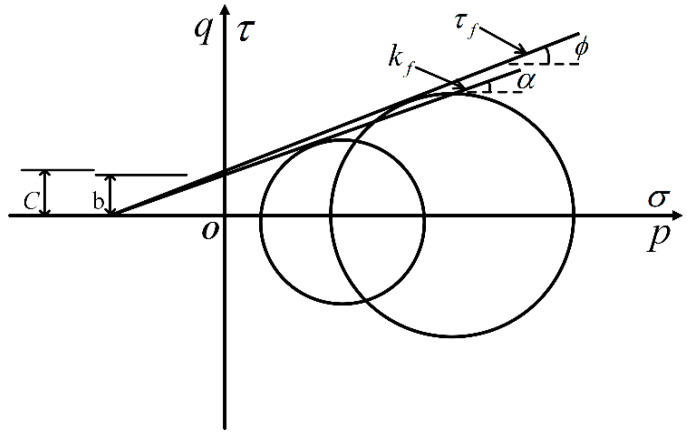
Illustration of $K_{f}$ line and $\tau_{f}$ line.

The line connecting ultimate stress domes under a variety of confined pressures is called the $K_{f}$ line, and the strength envelope is the $\tau_{f}$ line. The $K_{f}$ line is more convenient and accurate than the $\tau_{f}$ line to calculate shear strength parameters [50]. The relationship between the inclination α and intercept b of the $K_{f}$ line is related to the inclination ∅ and intercept c of the strength envelope $\tau_{f}$, as illustrated in Figure 11, and expressed as:

(4) ∅=arcsin(tanα),

(5) $$c=\frac{b}{\tan\alpha }\tan\emptyset =\frac{b}{\cos\emptyset },$$

where $K_{f}$ represents the ratio of principal stress in the case of failure:

(6) $$K_{f}=\frac{1-\tan\alpha }{1+\tan\alpha }=\frac{1-\sin\emptyset }{1+\sin\emptyset }=\tan^{2}\left(45{}^{\circ}-\frac{\emptyset }{2}\right),$$

**Figure 12:**
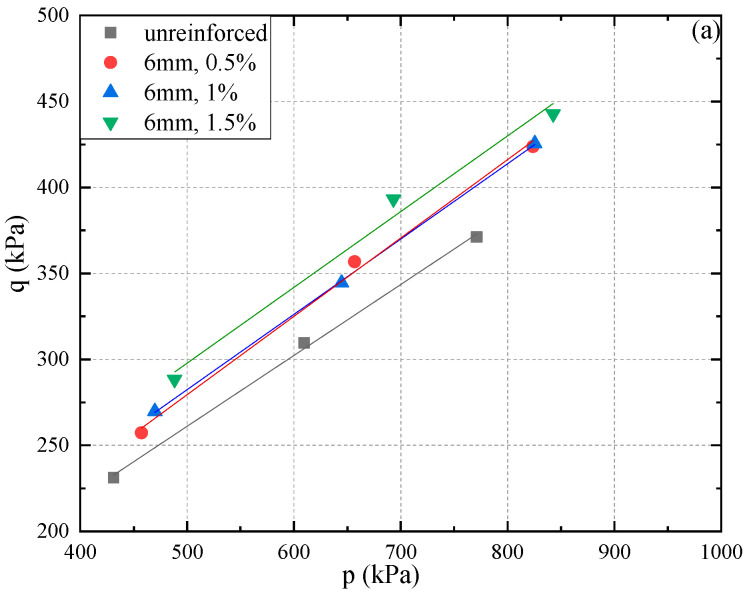

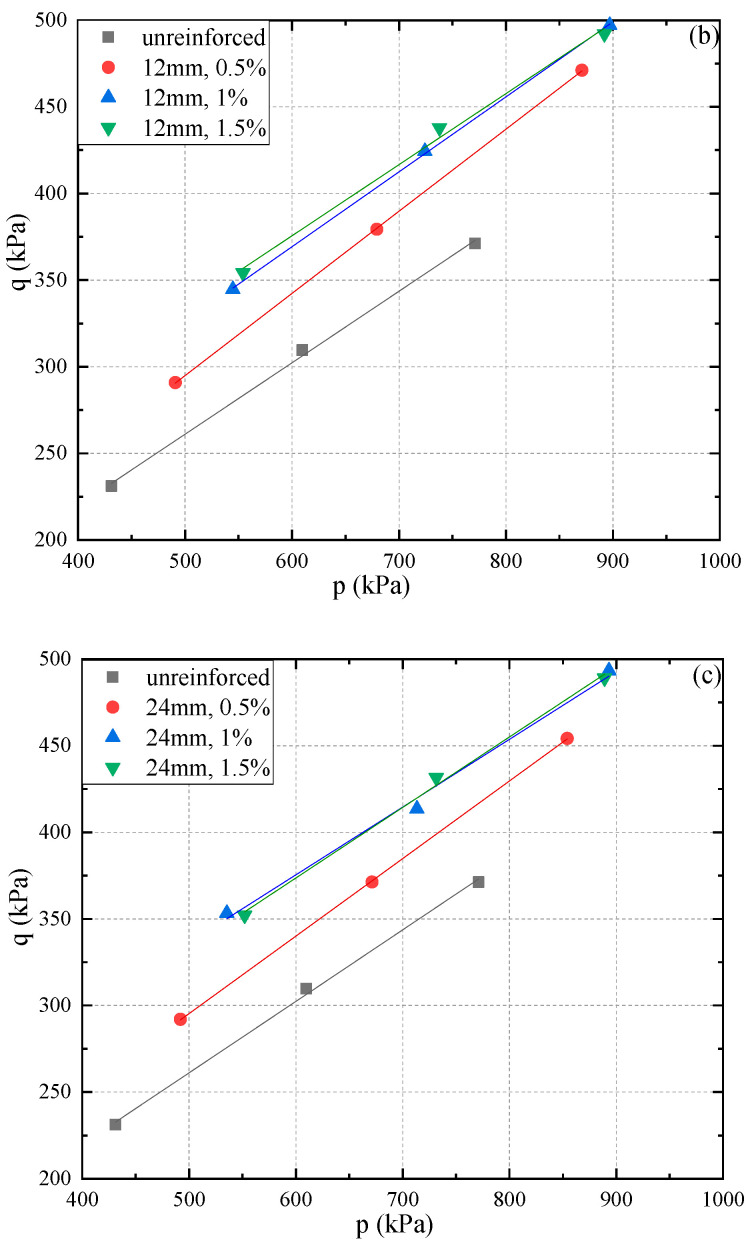
Lines for the polypropylene (PP) fiber-reinforced FAS specimens with various fiber lengths and fiber contents, (**a**) FC = 6 mm, (**b**) FC = 12 mm, (**c**) FC = 24 mm.

Figure 12 shows the $K_{f}$ lines for PP fiber-reinforced FAS specimens. With the rise of fiber content, it is found that the slope of the critical stress line for specimens with the same fiber length has a gradual increase. The slope of the critical stress line increases from 0.39 to 0.47 when the $K_{f}$ lines of non-reinforced FAS specimens are compared with those PP fiber-reinforced counterparts. In addition, it can be seen from the figure that the critical stress curves of PP fiber-reinforced FAS specimens are basically parallel to those of non-reinforced counterparts.

**Figure 13:**
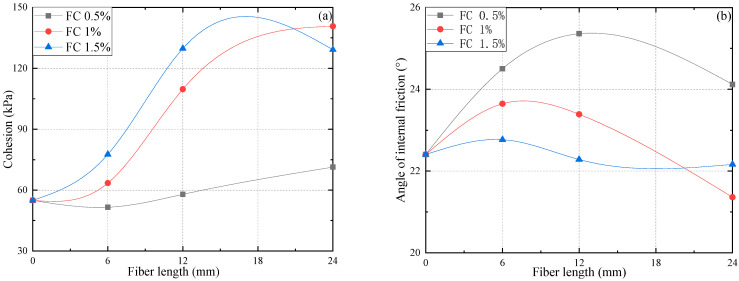
Shear strength parameters of reinforced specimens against various FL and FC, (**a**) cohesion, and (**b**) angle of internal friction.

Correspondingly, Figure 13 plots the φ and c of reinforced specimens with a variety of fiber lengths and fiber contents. It is observed that specimens with a particular fiber content have a significant increase in cohesion when fiber length increments from 0 mm to 12 mm. After that, the cohesion of FC 1.5% and FL 24 mm had a slight reduction. The maximum cohesion observed with FL 24 mm and FC 1% indicates that FC and FL considerably influence the cohesion of fiber-reinforced FAS specimens in the range of FC 1% and FL 12 mm; when exceeding the scope, a mild increase of the value could be verified.

Figure 13b indicates that the additional PP fiber results in a remarkable increase in the total shearing resistance angle of the specimen. The angle of internal friction shows a nonlinear change with the increment of FC and FL. Here, the reinforced specimens with FC 0.5% are taken as examples. The internal friction angles of these specimens show a variation from 24.5°, 25.4° to 24.1°, when FL increases from 6 mm to 24 mm, respectively. This finding is consistent with those of Wang et al. [30], i.e., that the angle of internal friction does not change in a linear way. However, it can be noticed that the total angle of shearing resistance of PP fiber-reinforced FAS specimens is insensitive to the transformation of fiber length.

### 3.6. The Energy Absorption Capacity

**Figure 14:**
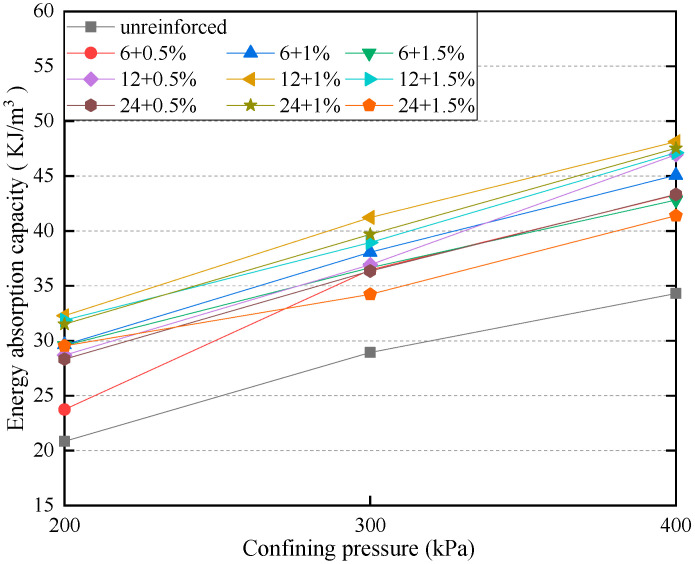
Variation of energy absorption capacity for the specimens boasting various FC and FL.

Figure 14 shows the variation in the energy absorption capacity of PP fiber-reinforced FAS specimens and non-reinforced FAS specimens.

It should be noted that the energy absorption capacity refers to the total sum of energy required to deform specimens during experiments and represents the ductility of PP fiber-reinforced FAS mixture specimens, which can be obtained through the calculation of the area beneath the stress–strain curve of specimens [51]. The higher the energy absorption capacity, the more difficult it would be to deform specimens. Figure 14 shows the energy absorption for an axial strain of 20% in all specimens. As a result, it can be clearly determined that the energy absorption capacity of specimens will considerably rise with the inclusion of fiber. Additionally, the energy absorption capacity will gradually increase as the confining pressure increments. The energy absorption capacity of reinforced soil with additional fibers is better than that of plain soil. As can be seen from the figure, energy absorption increased from 13.9% to 42.1% as FC developed from 0.5% to 1% when FL 6 mm was added; however, a reduction of energy absorption happened when FC changed to 1.5%. From Figure 14, we can see that the fiber length also has a certain impact on the reinforcement effect. Among specimens under all values of confining pressures, it can be found that the utmost energy absorption capacity is yielded when the FL is 12 mm and FC is 1%. It may be clarified that the residual strength of soil specimens can be improved by the incorporation of fibers. Meanwhile, the elongation of fibers enables specimens to absorb higher energy at a given axial strain.

## 4. Discussions

The inclusion of fly ash and PP fiber considerably enlarges the strength value of soils and obtains a ductile behavior after the FAS specimen failure. The reinforced stringiness behavior is able to convert the shear strength to a tensile strength while subjected to axial stress; therefore, the brittle failure of FAS changed into a ductile one that can stand prolonged residual strength.

Due to low calcium oxide content, unhydrated fly ash particles show irregular discrete distribution in the soil and are an insufficient binding gel between FAS particles. That may be the reason for unreinforced FAS specimen failure with a softening strain behavior as in plain soil. A considerable improvement appeared in the FAS specimen, even the 0.5% FC admixture of PP fiber. A similar phenomenon was also presented in these studies [16,37,46], in which physical friction occurred between FAS particles and PP fibers. FC and FL can both contribute increasingly to shear strength value, as in the low FC and FL reinforced FAS specimen, a fiber net failed to be constituted because of the major gap between fibers; as the FC and FL are going higher, FAS particles find it difficult to make contact with the fiber, and even a “floating” state is then present in fiber construction [46]. Displacement dislocation happened when subject to axial stress due to the elastic modulus of PP fiber being higher than the FAS specimen, leading to a non-consecutive deformation.

**Figure 15:**
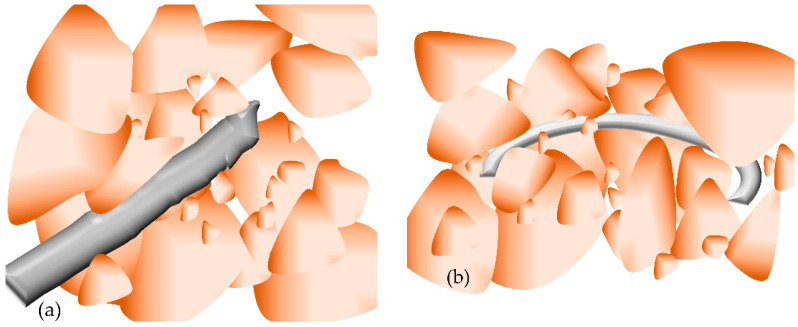

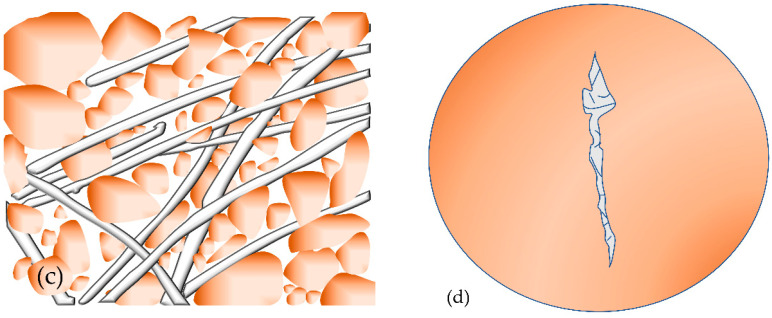
Fiber reinforcement mode, (**a**,**b**) one-dimensional reinforcement effect, (**c**) three-dimensional reinforcement effect, (**d**) “bridge” function.

The unreinforced FAS specimen had a more compact texture than plain soil, but the clear void was still present in the structure [4]. Wang et al. and Gao et al. [26,48] declared a microscopic binding force between soil particles and PP fibers due to discrete PP fiber distribution. Single fibers are squeezed and obvious plastic deformation is presented under stress load and tension, as fibers withstand tensile stress and resist the pullout force due to shear action. Figure 15a,b shows how one-dimensional reinforcement effects work on the PP fiber-reinforced FAS specimen; fiber/soil interaction and curved fibers wrapped soils in the bend and developed soil strength. Moreover, randomly discrete fibers interweave in the specimens; once the fiber is dislocated as a result of pullout force, adjacent fibers are moved to form a strong connection to redistribute the force area. The reinforcement structure behaving this way is mainly dominated by three-dimensional reinforcement action, as shown in Figure 15c. After the failure of the specimen, fibers distributed in the fractured surface will not break or pull out instantly, which is shown in Figure 15d, as reported by [17,33,52], who observed after splitting tensile tests a fiber “bridge” function that can enhance the durability and convert brittleness of FAS specimens.

## 5. Conclusions

In this study, the results of PP fiber on the mechanical properties of fiber-reinforced FAS mixture specimens were performed by UU triaxial tests. The main conclusions are as follows:

(1) A trend of transformation that FAS specimens convert strain softening to strain hardening is more noticeable when FC and FL get large. The tendency shows that the peak deviator stress hits the maximum value and then slightly changes as FL and FC increase. A significant consequence showed the optimum reinforced FL and FC will be 12 mm and 1%. It is clear that the axial stress of PP reinforced FAS increases at the peak of deviator stress. Shear strength and ductility develop with the increasingly confining pressure.

(2) Randomly oriented dispersed fiber incredibly developed FAS specimen failure strength with the increase of confining pressure. The deviator stress values of FL 6 mm with FC 0.5%, FC 1%, and FC 1.5% are 11.3%, 16.7%, and 24.7% higher than non-reinforced FAS under confining pressure of 200 kPa, respectively. The increase in strength value is due to the unobvious binding gel effect converting shear action into a tensile interaction between soil particles and fiber.

(3) The values of reinforcement coefficient $R_{\sigma }$ for all reinforced FAS specimens developed an upward trend, indicating that the fiber can lead to a significant improvement of the shear strength. The reinforcement coefficient decreases when FC increases from 6 mm to 12 mm. Among all values, 12 mm in fiber length and 1.5% in the fiber content are the optimum options with the highest value of $R_{\sigma }$.

(4) The variation of FC and FL increases the cohesion and changes the angle of internal friction of specimens. The maximum angle of internal friction occurs at FC 0.5% and FL 12 mm, resulting in a value 1.13 times higher than unreinforced FAS specimens. However, there is little impact on the cohesion of polypropylene (PP) fiber-reinforced FAS specimens by changing the length of the fiber, which may be due to the exceeded FC and limitation of specimen size.

(5) There is an apparent change within the energy absorption capacity of specimens while fiber is added. The maximum energy absorption capacity occurs when FL is 12 mm, and FC is 1%.

(6) The inclusion of fiber improved the brittleness behavior of soil by forming a “bridge” structure, and localized cracks were restricted and mobilized soil was stopped by admixture of fiber when specimens reached failure. The fiber reinforcement can reduce the loss of peak intensity and develop the residual strength in which one-dimensional and three-dimensional effects dominate the reinforced mode.

## Figures and Tables

**Table 1:** Physical properties of clay and fly ash.

Property	Clay	Fly Ash
Optimum moisture content, (%)	27.8	22.00
Natural moisture content, (%)	7.68	-
Natural density, (g/cm^3^)	1.35	2.16
$\mathrm{Specific}\;\mathrm{gravity},\;\;G_{s}$	2.68	2.16
Liquid limit, (%)	34.0	E4
Plastic limit, (%)	17.8	NP
Maximum dry density, (g/cm^3^)	1.42	1.36
Loss on ignition (LOI) (%)	-	2.74
Passing NO. 325 (45 μm) (%)	-	10.4

**Table 2:** Chemical composition of clay and fly ash.

Chemical Composition	SiO_2_	Fe_2_O_3_	Al_2_O_3_	CaO	MgO	SO_3_	K_2_O	Na_2_O	TiO_2_
Clay (%)	46.52	3.24	42.33	-	0.74	0.58	3.47	0.44	1.65
Fly ash (%)	60.62	6.85	22.35	1.96	1.16	0.16	2.46	0.50	1.20

**Table 3:** Mechanical properties of polypropylene (PP) fiber.

Fiber Type	Elastic Modulus (GPa)	Tensile Strength (MPa)	Elongation at Break (%)	Diameter (mm)
Polypropylene fiber	5.2	512	25	0.023

**Table 4:** Designation of the specimen.

Number	FAS (%)	FC (%)	FL (mm)	Curing Periods (d)
1	100	-	-	7
2	99.5	0.5	6	7
3	99	1	6	7
4	98.5	1.5	6	7
5	99.5	0.5	12	7
6	99	1	12	7
7	98.5	1.5	12	7
8	99.5	0.5	24	7
9	99	1	24	7
10	98.5	1.5	24	7
